# Klippel-trenaunay syndrome in a child with coexisting lymphangioma, vascular insufficiency, and multiple soft tissue swellings: a case report

**DOI:** 10.1093/omcr/omaf286

**Published:** 2026-01-25

**Authors:** Muhammad Abrar Amir, Muhammad Aniq Amir, Syed Ali Arsal, Saif Ullah Bin Bilal, Ahmed Ibrahim Siddiqui, Rameez Hussain, Oluwatobiloba Israel Popoola, Inibehe Ime Okon

**Affiliations:** Department of Medicine, Karachi Medical and Dental College, Block M North Nazimabad Town, Karachi City, Sindh 74700, Pakistan; Department of Medicine, Dow Medical College, Mission Rd, Nanak Wara Nanakwara, Karachi, Sindh 74200, Pakistan; Department of Medicine, Shaheed Mohtarma Benazir Bhutto Medical College, Lyari Hospital Rd, Rangiwara Karachi, Karachi 75100, Pakistan; Department of Medicine, Dow Medical College, Mission Rd, Nanak Wara Nanakwara, Karachi, Sindh 74200, Pakistan; Department of Medicine, Dow Medical College, Mission Rd, Nanak Wara Nanakwara, Karachi, Sindh 74200, Pakistan; Department of Medicine, Dow Medical College, Mission Rd, Nanak Wara Nanakwara, Karachi, Sindh 74200, Pakistan; Department of Medicine, Shenyang Medical College, Shenyang, Liaoning 110023, China; Department of Research, Medical Research Circle (MedReC), Bukavu 50, DR Congo

**Keywords:** Klippel-Trenaunay syndrome, Lymphangioma, capillary-venous-lymphatic malformation, vascular insufficiency, limb hypertrophy, capillary-venous-lymphatic malformation

## Abstract

**Introduction:**

Klippel-Trenaunay syndrome (KTS) is a complex and extremely rare congenital vascular syndrome. The disorder presents with a vascular malformation syndrome involving cutaneous capillaries and venous (hemangiomas and port-wine stains). Lymphatic anomalous development, with hyperplasia of soft tissue and bones, can also occur, which is due to overgrowth occurring as a result of somatic mutations

**Case Presentation:**

We present a 12-year-old male child with a 12-year history of subcutaneous growths, initially painless. KTS was diagnosed in childhood and associated with a vascular malformation of the right thigh, leg, and foot, associated with hypertrophy in the ipsilateral lower leg. Further, the patient had the presence of extensive lymphangioma in the right leg, which co-existed with the vascular malformation. The patient was treated with a multi-disciplinary approach

**Discussion:**

KTS is a congenital disorder characterized by varicose veins, capillary malformations, and tissue hypertrophy. It may show GI bleeding, orthopedic problems, and possible complications such as splenic hemangiomas. The disorder can be managed using NSAIDs, embolization, sclerotherapy, and surgery for severe cases. Genetic testing and imaging, including MRI, are important in diagnosis and management planning

**Conclusion:**

In conclusion, KTS is a challenging disease due to its rarity, diagnostic complexity, and varied clinical manifestations. It can be presented with different manifestations and complications, hence makes it very difficult for clinicians to diagnose. For the growing awareness, new research can enhance the knowledge of the clinician about the disease and provide better care for the individuals afflicted by KTS.

## Introduction

Klippel-Trenaunay syndrome or KTS is a complex and extremely rare congenital vascular syndrome that presents at birth, early infancy, or childhood. KTS was first described in the year 1900 in two patients with cutaneous hemangiomas associated with hypertrophy of soft tissue and bone [1]. It is estimated that KTS occurs in two to three cases per 100 000 live births, with no predilection for sex or ethnicity [2, 3].

In its classical form, the disorder presents with a vascular malformation syndrome involving cutaneous capillaries and venous (hemangiomas and port-wine stains), and lymphatic anomalous development, with hyperplasia of soft tissue and bones which is due to overgrowth occurring as a result of somatic mutations in the *PIK3CA* gene [4–6]. Although multiple extremities might be involved, KTS usually affects a single limb, with legs more commonly affected than arms. The limbs might become long due to the hypertrophy of all or one or two bones [7]. In rare cases of KTS, the affected limb may become paradoxically hypotrophic or shortened [6]. Such cases have been described as inverse Klippel-Trenaunay syndrome and are considered part of the syndrome’s spectrum [8, 9]. The cutaneous malformations are usually unilateral and involve the lower limb. Common complications of KTS include bleeding, deep vein thrombosis (DVT), and pulmonary embolism [10, 11].

Due to the varied nature of the symptoms, managing Klippel-Trénaunay Syndrome (KTS) necessitates a multidisciplinary approach and typically involves conservative treatment with lifelong monitoring to prevent complications. Definitive indications for intervention include hemorrhage, infections, acute thromboembolism, or persistent ulcers. Relative indications for treatment encompass pain, functional impairment, swelling from chronic venous insufficiency, limb asymmetry, and cosmetic concerns [12]. The symptomatic treatment involves addressing pain management through compression stockings, antibiotics, analgesics, and strict hygiene [13]. In advanced cases, surgery is deemed necessary.

## Case report

A 12-year-old male child presented to the pediatric emergency department with multiple swellings on the right leg and a fever. The fever was high-grade (documented: 101–102°F), intermittent, and not associated with rigors and chills, while the swelling was firm, smooth, warm, and associated with pain and tenderness. There was also a purulent discharge from the swellings that was yellow in colour. The patient also reports moderate pain in the leg around the swelling. Upon further questioning, we discovered that the child has a known case of Klippel-Trenaunay Syndrome and has a history of multiple hospital admissions in the past for the same reason. Right lower limb hypertrophy and vascular malformations have been evident on the right leg since birth and have been increasing with the child’s age. Vascular malformation and lymphedema were evident in the right leg clinically, which revealed the presence of extensive lymphangioma, emphasizing the vascular and lymphatic abnormalities that co-present in the disease.

**Figure 1:**
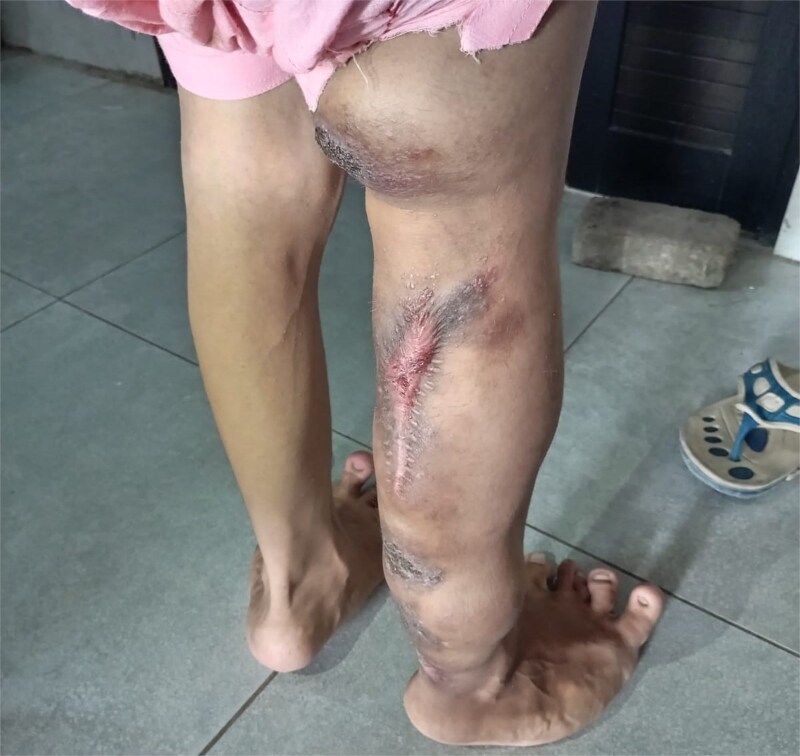
Patient’s right leg with lymphedema, visible vascular malformation and hypertrophic scar mark.

**Figure 2:**
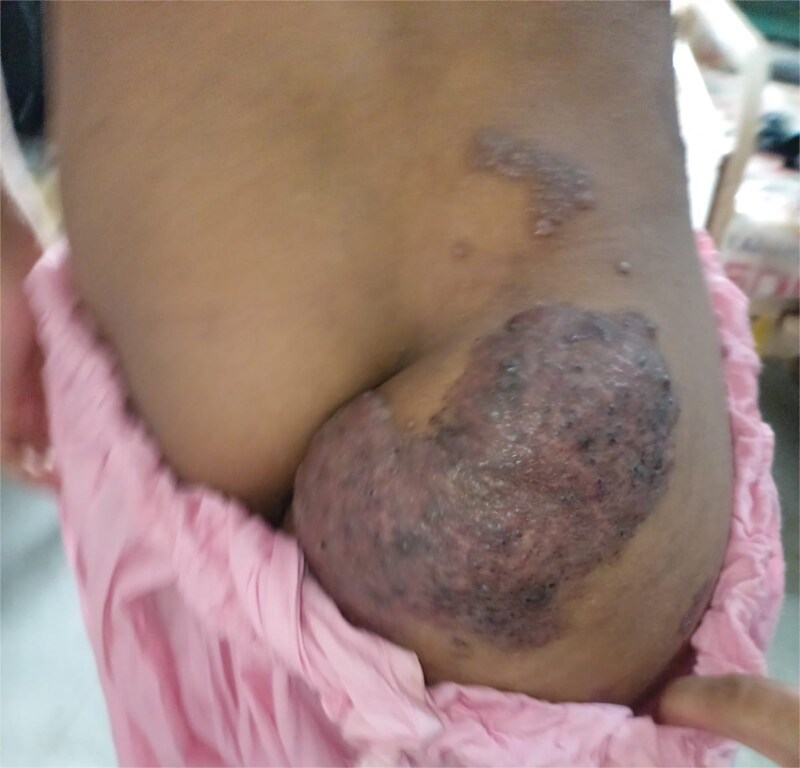
Visible hypertrophy and scaring at hip and gluteal region of the patient.

**Figure 3:**
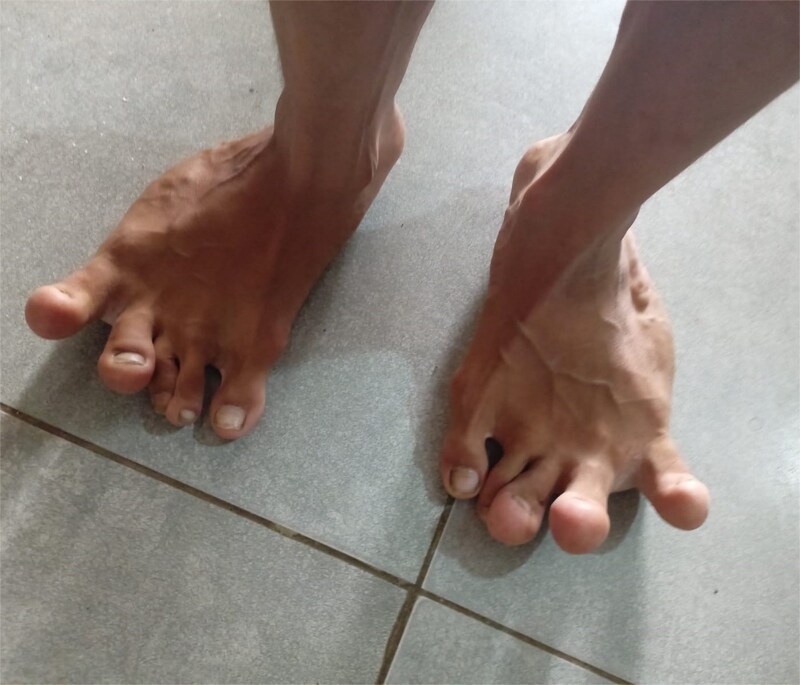
Visible hypertrophy of the feet and deviation of the toes.

On examination, the right leg was significantly hypertrophied, and there were multiple vascular malformations visible on the hip and dorsum of the right leg (Figs 1 and 2). The feet were bilaterally hypertrophied, with fingers and toes also involved. Moreover, the feet were club-shaped and flat (Fig. 3), and there was a large scar mark in the popliteal region due to the excision of previous drainage. Other than that, the veins on the right legs were engorged, but there weren’t any signs of vascular malformation or hypertrophy on the upper limbs and chest. We also performed his general physical exam and found no hypertrophy or distortion in the upper limb. Peripheral pulses were palpable, and the peripheries were warm, but pitting edema was evident in the lower limb. His abdominal, respiratory, and cardiovascular exam and central nervous system examinations were unremarkable. A motor examination of all limbs was also performed; the right leg was bulkier than the left, while the bulk was symmetrical in the upper limb. Power and tone were normal in all limbs, reflexes were intact and 2+, and planters were downgoing. The patient has a slight limp, but the lower and upper limbs were bilaterally equal in length. A summary of the patient’s clinical and unique features is presented in Table 2.

His lab investigations are shown in Table 1. Ultrasound Doppler of lower limb vessels involving the common femoral, superficial femoral, deep femoral, popliteal artery, anterior and posterior tibial, and dorsalis pedis vessels was performed, which showed moderate arterial insufficiency in the right lower limb arteries and mild to moderate insufficiency in the left limb. Soft tissue edema was also evident bilaterally, involving up to the dorsum of the feet, and there were no signs of thromboembolism.

**Table 1:** Baseline blood report of the patient.

Haematology	Electrolytes and acute phase reactant
Haemoglobin = 8.5 gm/dl	Sodium = 126 mEq/l
HCT = 25.4%	Pottasium = 3.5 mEq/l
MCV = 79.1 fl	Chloride = 91 mEq/l
MCH = 26.1Pg	Calcium = 6.7 mg/dl
Total Leucocyte Count = 34 000 cells/microliter	Creatinine = 0.8 mg/dl
Neutrophils = 91%	Blood Urea Nitrogen = 50 mg/dl
Lymphocytes = 5%	C-Reactive protein = 374.9 mg/l
Platelet Count = 534 000 cells/microlitre	

**Table 2:** Key features of the case presentations.

Clinical features	Key features
Age at presentation	12 years
Duration of symptoms	Since birth
Capillary malformation	Present – port-wine stain over right thigh and leg
Venous malformation	Present – varicosities and dilated veins in the right lower limb
Lymphatic involvement	Extensive lymphangioma co-existing with vascular malformation
Limb hypertrophy	Right thigh, leg, and foot
Pain	Initially, painless, mild discomfort with time.
Investigation:	Clinical presentation of capillary malformation, venous malformation, and limb hypertrophy. Ultrasound Doppler showing the right and left limb arterial insufficiency and soft tissue edema.
Management approach	Multidisciplinary – including pediatrician, surgeon, and dermatologist.
Distinctive aspect of the case	Coexistence of extensive lymphangioma with classical KTS features Use of sildenafil for the prevention of limb ischemia and lymphatic malformation.

During the hospital course, he received symptomatic fever treatment with paracetamol. He was commenced on an antibiotic course of ceftriaxone and vancomycin before he was referred to plastic surgery for excision and coverage of his swellings. We also took the dermatologist’s opinion for his multiple nodular swellings and ulcerated lesions, and were advised to apply fusidic acid and proper dressing with KMNO4-soaked gauze. To minimize edema and support his engorged veins, compression stockings were applied along with advice to elevate his leg when resting.

On follow-up, two weeks later, the patient was advised to continue the prescribed antibiotics. The plastic surgery team had performed excision and coverage of the swellings, after which the patient showed reduced local infection and improved wound healing.

He was also started on oral sildenafil 3 mg three times daily. In this patient, sildenafil administration resulted in subjective improvement in leg pain and better tolerance to ambulation, although over-the-counter analgesics were still required for pain relief.

## Discussion

Klippel-Trenaunay Syndrome (KTS) is a term used for a congenital disorder that includes varicose veins, cutaneous capillary malformation, and soft tissue or bone hypertrophy (sometimes involving the lymphatic system) [11]. The clinical presentation varies, and the patient can develop a spectrum of clinical findings, such as GI bleeding in approximately 20% of patients. The most common site of bleeding is the distal colon or rectum, and the spectrum of bleeding ranges from asymptomatic to massive bleeding that can be fatal [14, 15]. Orthopedic manifestation is present in almost one-third of patients in hypertrophy involving muscles, bones, soft tissue, and limb length discrepancy, most commonly [11, 16]. Hemangiomas, particularly involving the spleen, may occur as a part of generalized angiomas as KTS. Splenic hemangiomas can develop complications, including malignant degeneration and rupture, typically when they grow larger than 4 cm [15]. Our case showed port wine stain, which is pink to reddish-purple marks on the skin of his right leg and hip, which is the presentation of typical KTS, while the atypical type doesn’t present with port wine stain is rare.

In our case, we did a Doppler ultrasound scan to differentiate Klippel-Trenaunay-Weber syndrome (KTWS) from KTS. The features that help differentiate between the two on Doppler include venous abnormalities that in KTS show varicosities and venous malformation of superficial and deep veins of the affected limb, while in KTWS show arteriovenous malformation with arterialization of the venous system and blood flow characteristics that in KTS are mostly sluggish and in KTWS is high velocity and turbulent [17, 18]. Port wine stain is evident in Maffucci’s syndrome and Sturge–Weber’s, but no additional body tissue hypertrophy is visible. CLOVES (congenital lipomatous overgrowth vascular malformation with epidermal nevus and skeletal abnormalities) is also a mimicker of KTS with involving both. Cutis marmorata telangiectasia congenita, hemi hyperplasia, and numerous lipomas. Unlike KTS, it often presents with truncal involvement and complex lymphatic anomalies. Beckwith-Wiedemann syndrome and hemihyperplasia syndromes exhibit asymmetric overgrowth but lack the complex vascular anomalies of KTS. Servelle-Martorell syndrome is marked by bone hypotrophy associated with extensive venous malformations, in contrast to the hypertrophy seen in KTS. Proteus syndrome presents with asymmetric overgrowth, cerebriform connective tissue nevi, and disproportionate limb enlargement, but lacks the classical port-wine stains of KTS [19].

Ultrasound Doppler is the first-line investigation for assessing venous and arterial malformations in KTS. In our case, Doppler confirmed low-flow venous malformations and revealed arterial insufficiency, which influenced the management decision. Advanced imaging, such as magnetic resonance imaging and computed tomography [20], is of particular importance in identifying the extent of vascular malformation and guiding surgical planning. Genetic testing for detecting somatic mutations in the PIK3CA can guide the use of therapies such as PI3K inhibitors (e.g. alpelisib) in refractory or complex cases [21]. Unfortunately, advanced imaging and genetic analysis were not available in our setting, representing a limitation in the comprehensive evaluation of this patient.

A multi-disciplinary team approach with close coordination is required when caring for a patient with such complex diseases involving an orthopedic surgeon, vascular surgeon, dermatologist, general physician, and general surgeon. Medical management of these patients involves NSAIDs, analgesics, and diuretics. Embolization and sclerotherapy are used for treating vascular malformation, and surgical resection is only reserved for those whose vascular malformation didn’t respond to embolization and vascular therapy. Laser therapy can be offered for port-wine stain, and limb length discrepancy requires orthopedic intervention [5].

In our case, the patient was managed conservatively with oral analgesics for pain management. Intravenous antibiotics (Ceftriaxone and Vancomycin) were started for infected lymphangiomas, along with local wound care, compression therapy, and leg elevation. Oral sildenafil was prescribed, which, in recent research, has been explored as a potential treatment for complex lymphatic malformation [22]. Sildenafil is a phosphodiesterase type 5 inhibitor that prevents the breakdown of cyclic guanosine monophosphate (cGMP), leading to smooth muscle relaxation and vasodilation [23]. Sildenafil can promote vasodilation, angiogenesis, reduce oxidative stress, and improve blood flow, thereby relieving symptoms such as pain and ischemia associated with the syndrome [24, 25].

The limitation of this study includes the genetic testing and MRI with contrast of the affected limb, as these are typically used to provide deeper insights into etiology, potential hereditary factors, and detailed vascular anomalies associated with KTS. Moreover, CT is also recommended, especially in patients with renal involvement and suspicion of pulmonary embolism, which is quite common in the disease, but wasn’t performed in our case. While a biopsy of the affected area is considered the gold standard for the diagnosis of lymphangioma, it was clinically diagnosed in our case. However, these limitations can serve as strengths while dealing with KTS syndrome in an underserved healthcare setting.

## Data Availability

Not applicable.
